# Genetic investigation of non-affective psychosis and depression as causal risk factors for dementia

**DOI:** 10.1136/bmjment-2025-302424

**Published:** 2026-06-24

**Authors:** Valentina Escott-Price, Emily Simmonds, Michael J Owen, Michael O’Donovan

**Affiliations:** 1 Division of Psychological Medicine and Clinical Neurosciences, Cardiff University, Cardiff, UK; 2 UK Dementia Research Institute at Cardiff University, Cardiff, UK

**Keywords:** Dementia, Depressive Disorder, Mental Health, Mood Disorders, Psychotic Disorders

## Abstract

**Background:**

Major psychiatric disorders are associated with increased risk of dementia but establishing whether psychiatric disorders causally increase dementia risk is challenging because dementia pathology can precede clinical diagnosis by decades. Prodromal psychiatric symptoms may arise long before cognitive decline, leaving open the possibility of reverse causation.

**Objective:**

We aimed to determine whether non-affective psychosis or depression credibly causally influence dementia risk using a design robust to reverse causation. We tested whether people with psychiatric disorders who later develop dementia show reduced genetic liability to Alzheimer’s disease (AD) compared with dementia cases without such history.

**Methods:**

We compared AD genetic liability, measured by polygenic risk scores (PRS) among dementia cases (N=7936) with and without prior non-affective psychosis (N=56) or depression (N=937) in the UK Biobank. We examined whether schizophrenia or major depressive disorder (MDD) PRS correlates with dementia liability to assess whether shared trait liability contributes to the association.

**Findings:**

Dementia cases with prior non-affective psychosis or depression had lower AD genetic liability than those without a psychiatric history (psychosis: B=−0.29, 95% CI (−0.54 to −0.05), p=0.036; depression: B=−0.12, 95% CI (−0.18 to −0.05), p=0.0004), which is inconsistent with the hypothesis that the association between psychiatric disorders in dementia is explained by prodromal dementia effects. After excluding people with psychiatric diagnoses, neither schizophrenia nor MDD liability showed the negative correlations with AD liability in people with dementia expected if trait liability to those disorders per se contributed to dementia risk. Our findings instead are consistent with exposure to the disorders themselves as being associated with dementia.

**Conclusions:**

Our findings are consistent with the hypothesis that psychiatric disorders are associated with increased vulnerability to dementia.

**Clinical implications:**

Identification of potentially modifiable mechanisms for the association and optimal management of non-affective psychosis and depression may help reduce long-term dementia risk and inform prevention strategies.

**WHAT IS ALREADY KNOWN ON THIS TOPIC**

- People with psychiatric disorders, especially schizophrenia, other psychotic disorders and depression, show elevated dementia risk. Non-affective psychosis (schizophrenia, delusional disorder, acute and transient psychotic disorders, schizoaffective disorder, unspecified nonorganic psychosis) is associated with a threefold to fourfold increase in risk, while in depression, the increased risk is up to twofold. Because dementia pathology begins before cognitive change, psychiatric symptoms may reflect prodromal dementia, complicating causal inference.

**WHAT THIS STUDY ADDS**

- Using a design robust to reverse causation, we found that people with prior non-affective psychosis or depression showed lower Alzheimer’s liability than those with dementia alone, consistent with a causal role of psychiatric disorders rather than prodromal effects. Follow-up analyses indicated little evidence for shared genetic liability, indicating that exposure to psychiatric disorders themselves increases subsequent dementia risk.

**HOW THIS STUDY MIGHT AFFECT RESEARCH, PRACTICE OR POLICY**

- This highlights psychiatric disorders as potential causal risk factors for dementia. It underscores the need for early monitoring and dementia-prevention strategies in patients with psychiatric disorders. Further research is required to identify potentially modifiable mechanisms for the association. Policymakers may consider integrating psychiatric care with dementia risk assessment and long-term prevention planning.

## Introduction

### Background

 Higher rates of dementia have been associated with several psychiatric disorders, particularly psychotic disorders and depression.^1 2^ We and others reported that schizophrenia and related non-affective psychotic disorders are associated with a 3–4-fold increased risk of dementia^2 3^ and that depressive disorders with around a 1.6–2-fold increased risk.^3^

Dementia pathology can occur at least 10–20 years before a clinical diagnosis of dementia^4^ and may result in prodromal psychiatric symptoms.^5 6^ Psychiatric symptoms may also arise after dementia onset.^2^ This makes establishing the direction of causality responsible for the association between psychiatric disorders and dementia complicated even with longitudinal data. Studies including our own^2^ have sought to minimise the impact of prodromal psychiatric symptoms caused by dementia by excluding individuals where the psychiatric disorders are first recorded a few years prior to that of dementia^7^ but even this cannot completely address the possibility of reverse causation since it is unclear how early prodromal psychiatric symptoms may occur. Thus, it has been reported that psychiatric symptoms may predate a clinical diagnosis of dementia by up to 5 years^8^ while another study^9^ reported mental distress to be associated up to 27 years later with increased risk of dementia (~1.5-fold increase). Illustrating the challenge, the authors of the latter study noted they were unable to exclude the possibility that this association might still reflect prodromal manifestations of dementia.

The relationship between psychiatric conditions and the risk of dementia is complex. It depends on the type, severity and age at onset of the psychiatric condition, each of which may influence the type of dementia that develops.^10^ Our earlier study^2^ showed that the first onset of psychiatric disorders is highest in the year preceding the dementia diagnosis, but also progressively over several preceding years, with rates gradually decreasing the further from dementia diagnosis. It is likely that studies with limited follow-back periods underestimate the risk associated with psychiatric conditions. Moreover, if prodromal psychiatric manifestations of neurodegeneration are manifest many years before dementia onset, then reverse causation could lead to an overestimate of causal effects. On the other hand, if researchers ‘overcorrect’ by excluding onsets too far back, then this will underestimate causal effects.

Estimates genetic correlation between Alzheimer’s disease (AD) and schizophrenia spectrum disorders are consistently low.^11^ A significant positive genetic correlation was observed between depression and AD in a genome-wide association study (GWAS).^12^ However, this included cases of AD defined by proxy through family history, which has been criticised,^13^ and no significant genetic correlation between this disorder was detected in a sample without proxy cases.^14^ Mendelian randomisation (MR) studies are consistent with a weak causal effect of schizophrenia on broadly defined dementia^15^ and between depression and vascular dementia (VaD).^16^ However, caution is required in inferring causality based on MR alone as it is often unclear that the assumptions required by MR are met.^17^ Moreover, the published studies have relied on GWAS summary statistics precluding stratification by age at diagnosis. Where individual-level genotype and phenotype data are available for stratified analyses polygenic risk scores (PRS) offer a complementary approach being better powered than MR in modest target samples.^18^

### Objective

Here, we test the hypothesis that there are causal associations between dementia and two sets of disorders, non-affective psychosis (schizophrenia, delusional disorder, acute and transient psychotic disorders, schizoaffective disorder, unspecified nonorganic psychosis) and depression, using a study design that is robust to reverse causation. If psychiatric disorder causally increases the risk of dementia, it follows from the liability threshold model of complex disorders that individuals with that psychiatric disorder who later develop dementia will require fewer independent additional dementia risk factors, including a lower burden of genetic liability to dementia, than those without that psychiatric disorder who develop dementia. However, if the association can be explained by prodromal effects of dementia pathology on psychopathology, people with psychiatric disorder and dementia would not be expected to have a lower burden of dementia risk factors than people with only a diagnosis of dementia.

If psychiatric disorders indeed increase risk of dementia, this poses the question as to whether this reflects a shared underlying pathophysiology, or whether dementia is attributable to the manifestation of disorders themselves and/or to exposures that are secondary to them. If the underlying psychiatric pathophysiology has a causal effect on dementia, assuming that the severity of an individual’s underlying psychiatric pathophysiology correlates with genetic liability to that psychiatric disorder, liability to the psychiatric disorder per se should be a dementia risk factor even in those who do not have the relevant psychiatric disorder. People with dementia at higher genetic liability to the psychiatric disorder would then require lower genetic liability to dementia, even those who do not manifest the psychiatric disorder. Liability to psychiatric disorder and to dementia should then be negatively correlated in people with dementia and this would be true in those without the relevant psychiatric disorder.

Here, we test these hypotheses in the UK Biobank (UKB)^19^ using PRS as measures of genetic risk for AD, schizophrenia representing non-affective psychosis and major depressive disorder (MDD).

## Methods

### Dataset

The UKB comprises around half a million volunteers from the UK recruited between 2006 and 2010 when they were between 40 and 70 years of age.^19^ Participants were approached by mail and those who consented to take part were recruited. Our study used data under approved UKB application #109607. Diagnoses of dementia, psychotic disorder or depression (table 1, online supplemental table 1) were according to ICD-10 codes based on information from hospital admissions, death certificates, self-reported conditions and primary care records as provided by the UKB under category 1712 ‘First occurrences’. This category provides the date of the first report of any ICD-10 codes based on all the above information from which we derived each individual’s age at first report (AAFR). Diagnoses made before the recruitment point were also accepted. Actual age was calculated as difference between 2024 and the year of birth (UKB code 33) or age at death (UKB code 40007), where relevant.

**Table 1:** Sample demographics

	N	Age in 2023 (SD)	N dead	Age at death (SD)	Age at first report (SD)
Dementia	7936	78.8 (5.1)	4039	67.8 (8.7)	74.6 (6.0)
Alzheimer’s disease	3657	79.4 (4.5)	1849	68.1 (8.6)	75.4 (5.2)
Vascular dementia	1765	79.4 (4.4)	1042	67.7 (8.7)	75.4 (5.1)
Parkinson’s disease with dementia	1103	78.5 (4.7)	747	67.6 (8.5)	70.7 (8.1)
Unspecified dementia	2187	77.8 (6.0)	948	67.7 (8.8)	73.8 (7.5)
No dementia	395 173	71.3 (8.0)	31 266	65.3 (8.9)	NA
Non-affective psychosis	1874	69.9 (8.3)	471	65.5 (8.6)	52.5 (17.7)
Depression	52 439	70.3 (7.9)	6230	66.3 (8.9)	49.9 (15.6)
No psychiatric diagnosis*	330 501	71.5 (8.0)	26 777	65.4 (9.0)	NA

People with multiple diagnoses are not excluded.

* People with non-affective psychosis, bipolar disorder, depression and anxiety were excluded.

We included as dementia cases people with diagnoses of AD, VaD, Parkinson’s disease (PD) with dementia and unspecified dementia, the latter defined excluding AD, VaD, PD, amyotrophic lateral sclerosis and frontotemporal dementia (online supplemental table 1). We excluded people with a primary diagnosis of amyotrophic lateral sclerosis and frontotemporal dementia^20 21^ as there is evidence they show non-trivial genetic correlations with schizophrenia and dementia thought to be secondary to multiple sclerosis.

We assigned a classification of non-affective psychosis to people with a record of schizophrenia, delusional disorder, acute and transient psychotic disorders and schizoaffective disorder, and a classification of depression with diagnoses of single depressive episode, recurrent depressive episode, persistent mood affective disorders and unspecified mood disorder. The ‘No Psychiatric diagnosis’ group excluded people with non-affective psychosis, bipolar disorder, depression and anxiety (see online supplemental table 1 for ICD-10 codes). We excluded those who were most likely to have symptomatology secondary to dementia based on an AAFR of the relevant psychiatric disorder in the same year as, or, in the years after, the diagnosis of dementia, or whose AAFR of psychiatric disorder >65. This cut-off was selected since psychiatric disorders generally have an early age at onset and as a result, as their AAFR increases, either the deviation between true age at onset and AAFR is likely to increase and/or we would increasingly include people whose true age at onset occurs within the prodromal range of dementia or even after the age at onset of dementia.

### PRS calculation

PRS for MDD and schizophrenia were computed using summary statistics^22^ and^23^ respectively, downloaded from the Psychiatric Genetics Consortium website, selecting at the time of download (October 2023) the most recent GWAS in people of European ancestry. AD summary statistics^24^ were used for the AD-PRS calculations, as it is the latest publicly available AD GWAS that does not include UKB participants. We limited analyses to UKB participants of White British or Irish ancestry, as PRS relies on SNP effect sizes best estimated in European populations.

We used PRS-CS^25^ to adjust GWAS effect sizes for Linkage Disequilibrium (LD), with default settings and data-optimised shrinkage. LD was estimated using the UKB reference panel. Adjusted weights were applied in PLINK-v2^26^ to compute PRS. UKB imputed data underwent QC (INFO<0.4, MAF ≤1%, missingness >5%, HWE p <1e-6) and remaining SNPs were included. PRS were adjusted for 15 ancestry principal components and standardised.

### Statistical analysis

We tested whether among people with dementia, those who had AAFR for non-affective psychosis or depression below 65 and at least a year prior to that for dementia, had lower AD-PRS than in the dementia only group. We used logistic regression to test associations between AD-PRS and dementia risk in individuals with non-affective psychosis or MDD (vs dementia only), adjusting for sex. Age was not included since all participants had dementia. Pearson’s correlation assessed associations between AD-PRS and psychiatric disorder PRS.

We explored possible mediators of disorder on risk of dementia by adjusting our primary analyses for a number of dementia risk factors that are also associated with psychiatric disorder: smoking, body mass index (BMI), addiction to alcohol, addiction to any substance or behaviour, hypertension and educational attainment, which were included as ‘factor’ in the regression model (see online supplemental table 2 for UKB codes).

We report p values with correction for testing two primary hypotheses: (1) comparison of AD-PRS in people with dementia and a pre-existing diagnosis (AAFR≤65) of non-affective psychosis versus those with dementia alone and (2) comparison of AD-PRS in people with dementia and a pre-existing diagnosis of depression (AAFR≤65) versus those with dementia alone. Other sensitivity tests aimed to explain the primary findings (ie, conditioning on environmental exposures, tests of genetic correlation).

## Results

The initial number of people included in this study was 408 633 (UK Biobank code 22006: ‘Caucasian’) of whom 8294 had diagnosis of dementia by 2023. After exclusion of people with amyotrophic lateral sclerosis, frontotemporal dementia, multiple sclerosis and those with PD without dementia, we retained 7936 and 395 173 people, respectively, with and without dementia. Of the 7936 individuals, 3657, 1765 and 1103 people had diagnoses of AD, VaD and PD with dementia, respectively (table 1). Some individuals had more than one diagnosis.

### Non-affective psychosis and dementia

There were 1874 people with non-affective psychosis with mean AAFR of 52.5 years (table 1), of whom 205 (10.9%) also had a diagnosis of dementia. After exclusions based on AAFR (the Methods section) for non-affective psychosis, we retained 56 and 7731 people with/without psychosis, respectively, for our primary analysis.

**Figure 1:**
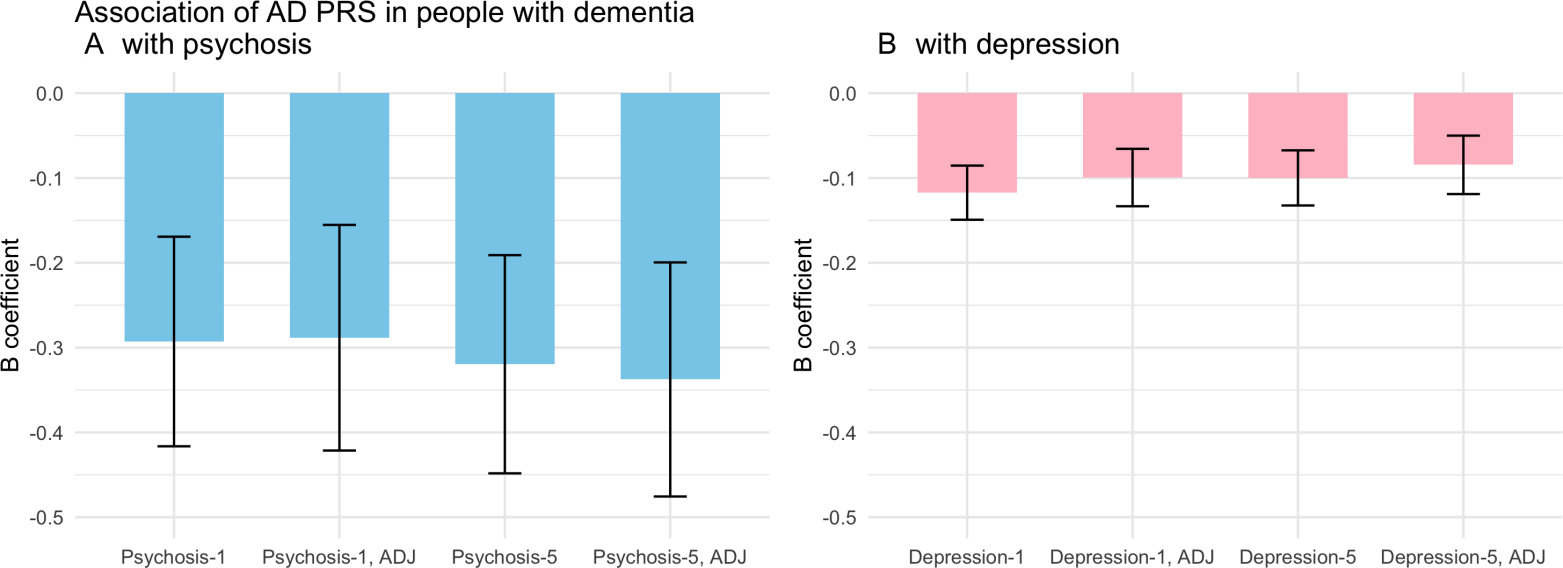
Association of AD PRS with non-affective psychosis (**A**) and depression (**B**) (AAFR≤65) in people with dementia. ‘Psychiatric disorder-1’—AAFR of psychiatric disorder at least 1 year before the AAFR of dementia; ‘Psychiatric disorder-5’—AAFR of psychiatric disorder at least 5 years before the AAFR of dementia; ‘Psychiatric disorder-1, ADJ’ and ‘Psychiatric disorder-5, ADJ’, as above but also adjusted for smoking, alcohol and substance addiction, hypertension, BMI and highest educational level achieved. AAFR, age at first report; AD PRS, Alzheimer’s disease polygenic risk scores; BMI, body mass index.

The AD-PRS was lower in the group with psychosis compared with those with dementia but not non-affective psychosis (B=−0.29, 95% CI (−0.54 to −0.05), p=0.036). Similar results were obtained (B=−0.32, 95% CI (−0.57 to −0.07), p=0.013) in a sensitivity test in which we excluded people with a AAFR diagnosis of non-affective psychosis <5 years prior to that for dementia (N=52 in analysis) figure 1A. As a sensitivity analysis, we reran these models using multiple AAFR cut-offs for non-affective psychosis (see online supplemental table 3A). The effect sizes for the association with AD PRS were consistent across all cut-offs. We also reanalysed the data excluding dementia cases from the comparator group with any psychiatric diagnosis (N=5057). The results were slightly more significant (online supplemental table 3B).

### Depression and dementia

The number of people with a depression diagnosis was 52 439, with mean AAFR of around 50 (see table 1), of whom 2142 (4%) had a dementia diagnosis. After exclusions based on AAFR (the Methods section) for depression, we retained 937 and 5794 people with/without depression for our primary analyses. The AD-PRS was lower in people with dementia and depression (B=−0.12, 95% CI (−0.18 to −0.05), p=0.0004) than those with dementia but no record of depression. A sensitivity test excluding people with an AAFR diagnosis of depression less than 5 years prior to that for dementia gave similar results (B=−0.10, 95% CI (−0.16 to −0.04), p=0.0019) (figure 1B). Analyses using multiple AAFR cut-offs for depression gave effect sizes for the association with AD PRS consistent with the primary analysis (online supplemental table 3A). Excluding from the comparator group those with dementia who had any other psychiatric diagnosis (N=5057) gave similar, although marginally more significant, results (online supplemental table 3B).

### Liability to schizophrenia and depression

In the whole Biobank sample, PRS-AD was weakly but significantly positively correlated with PRS-schizophrenia (r=0.020, p=2.2×10^-37^) and PRS-MDD (r=0.005, p=0.0009). After adjusting for diagnosis of non-affective psychosis and sex, SZ-PRS was not significantly associated with dementia (B=0.017, 95% CI (−0.00 to 0.04), p=0.124). MDD-PRS was, however, very weakly positively associated with dementia (B=0.026, 95% CI (0.00 to 0.050), p=0.022), adjusting for sex and the psychiatric diagnoses.

In those with dementia but not non-affective psychosis, we did not see the negative correlation between PRS for schizophrenia and PRS for AD that would be predicted if liability to non-affective psychosis rather than non-affective psychosis itself was associated with increased risk of dementia (r=0.007, p=0.558). PRS for AD and MDD did show some evidence for a weak negative correlation in people with dementia but without depression, however this was not significant (r=−0.013, p=0.312). Results were similar when including cases with AAFR ≤65 (r=0.006, p=0.568; r=−0.016, p=0.156, for non-affective psychosis and MDD, respectively).

### Mediators and confounders

Alcohol addiction and addiction to any substance or behaviour were reported by 0.73% and 1.6% of participants, respectively. As a proxy for cardiovascular problems, we used hypertension, which was present in 40.5% of UKB participants. The distribution of confounders across subgroups of individuals with psychosis, depression, no psychiatric diagnosis and with or without a dementia diagnosis is presented in online supplemental table 2 and online supplemental figure 1.

Here, we tested in individuals with dementia whether in the subgroup with antecedent psychiatric diagnoses, the AD-PRS remains lower compared with those without these prior psychiatric diagnoses, despite shared confounding factors or mediators between psychiatric disorders and dementia. After adjusting our primary analyses for smoking, BMI, hypertension, educational attainment, alcohol and substance addiction, the AD-PRS remained significantly lower in people with non-affective psychosis or depression with the effect sizes minimally changed from the primary analyses (B=−0.29, 95% CI (−0.55 to −0.03), p=0.031 and B=−0.10, 95% CI (−0.17 to −0.03), p=0.0033, respectively, see figure 1A,B).

The distribution of subtypes of dementia occurring in people with non-affective psychosis or with depression with age at first record below 65 years was not different from that in the population without these diagnoses (see online supplemental tables 3 and 4).

## Discussion

Here, we aimed to determine whether non-affective psychosis or depression confers risk for dementia using a study design that is robust to reverse causation. Our primary analysis revealed that in dementia, people with a history of non-affective psychosis or depression have lower genetic liability to AD than people without such a psychiatric history. This is inconsistent with the hypothesis that the elevated rates of psychiatric disorders in people who subsequently develop dementia reflect prodromal symptoms of dementia but is consistent with the hypothesis of increased vulnerability to dementia in people with, or higher liability to, psychiatric disorders. Confidence in this conclusion is elevated by the fact that inclusion of people with psychiatric symptoms that are prodromal would favour type 2, not type 1 error.

Follow-up analyses showed that liability to schizophrenia was weakly correlated with liability to AD, but dementia was not associated with liability to schizophrenia after adjusting for sex and for a history of non-affective psychosis. Moreover, in people with dementia but no history of non-affective psychosis, we did not see the inverse correlation between liability to AD and that for schizophrenia expected if liability to schizophrenia (rather than the disorder itself) conferred risk of dementia. After adjusting for sex and a history of depression, there was evidence for a weak association (B=0.026) between liability to MDD and dementia, but in people with dementia, but no history of depression, we did not find a significant negative correlation in liability to MDD and dementia. These findings imply that the association between the psychiatric disorders and dementia cannot be attributed to shared trait liability to both sets of disorders. Instead, they are consistent with exposure to the disorders themselves being associated with increased liability to dementia.

Note that by excluding participants whose AAFR of dementia predates the AAFR of psychiatric disorder, estimates of effect size do not generalise to all people with psychiatric disorder, therefore our study cannot provide epidemiological estimates of the impact of psychiatric disorder on the risk of dementia at a population level, although we would speculate that this impact is greatest for those with earlier onset of psychiatric disorder.

Since this study was initiated, others published a study of the UKB^5^ that examined the relationship between psychiatric disorders and dementia. While this used a different analytic methodology, the findings were also consistent with the conclusion that the psychiatric disorders-dementia association is not explained by prodromal effects of dementia. However, the authors suggested that the link is unlikely to be explained by a causal role of psychiatric disorders in dementia, although they also noted such a role cannot be excluded. This suggestion was based on a weak negative correlation between predementia psychiatric disorders and AD-PRS in dementia cases.

A key difference between the studies is that we focused on relatively specific psychiatric disorders, treating each separately, whereas the previous study included a broad set of disorders (representing about 20% of the UKBB). Our effect sizes were stronger (B=−0.29 for non-affective psychosis, B=−0.12 for depression compared with their regression coefficient=−0.07, p=0.002). We have also confirmed our findings excluding people with non-affective psychosis/depression 5 years prior to dementia diagnosis.

We cannot distinguish between direct effects of the psychiatric disorders on dementia, for example, changes in neuronal number or function that are contingent on manifesting the disorders but do not reflect liability to them, or whether the psychiatric disorders increase risk indirectly. Such indirect factors might include treatment of the disorders or increased exposure to environmental factors, for example, smoking, recreational drug usage, poor nutrition, low levels of exercise and social isolation.^27^ We did explore a limited number of risk factors for dementia that are more common in people with major psychiatric disorders but adjusting for these exposures did not substantially attenuate the effect sizes of our primary analyses, suggesting these are unlikely to mediate the association between the psychiatric disorders and dementia or represent shared risk factors that account for the association between the two sets of disorders.

There is a possibility that psychiatric conditions are causal for a specific subtype of dementia. For example, a retrospective study showed that participants with late-life depressive symptoms had a twofold increase in AD risk, whereas subjects with midlife and late-life symptoms had more than a threefold increase in VaD risk.^28^ Combining subtypes of dementia into one category as we have done in our primary analyses may reduce the power of the analyses. However, although we have limited power to test whether the distributions of types of dementia differ between those with and without non-affective psychosis, we found no evidence that they differed in the larger sample of participants with depression. We tested whether the subtypes of dementia occurring in people with non-affective psychosis or with depression and found that the patterns of association were the same as in the group of all dementia cases, in particular, those with AD and a psychiatric condition had a lower AD-PRS than those without. These findings suggest the lower dementia risks in people with additional diagnoses of non-affective psychosis or depression do not reflect association between the psychiatric disorders with forms of dementia with relatively low AD-PRS that might be especially prone to cause early prodromal psychiatric symptoms.

Our study has several limitations. First, the sample size for our analyses of psychosis was small, although that for depression was well within the range of well-powered studies exploiting polygenic scores since statistical power is not only a function of the number of ‘cases’ but of the total sample, which in the present study is the number of people with dementia (N=7936). The consistency of the findings across psychosis and depression strengthens confidence that the results are unlikely to reflect type 1 error. Crucially, as our primary findings do not depend on interpreting failures to find associations, they cannot be attributed to type 2 error.

Second, the accuracy of the diagnoses of dementia and psychiatric conditions is not perfect. This may decrease the power of the analyses. Separating the ‘dementia’ category into subtypes (AD, PD, VaD), the sample sizes of these subgroups with psychiatric conditions, particularly non-affective psychosis, were small limiting the power of that secondary analysis.

Third, the electronic records integrated into the UKB date back to 1997, 1998 and 1981 for England, Wales and Scotland, respectively. Given psychiatric disorders typically manifest for the first time in early adulthood, the effect of electronic records starting in later years means that the AAFR of psychiatric diagnoses derived from these records will typically be greater than the true age at onset of these conditions. We, therefore, regard AAFR of psychiatric disorder for an individual as the maximum age at onset rather than the actual age at onset. For the present study, the effect of these inaccuracies in specifying a true age at onset would result in loss of power due to the erroneous exclusion of people whose records indicate psychiatric disorder has occurred around the time of, or indeed after, that of dementia, and loss of power due to the erroneous inclusion of people whose dementia genuinely occurred before the onset of psychiatric disorder. A strength of our design is, however, that neither effect will increase the risk of a false-positive finding consistent with a causal effect of psychiatric disorder on dementia.

Fourth, while we sought in secondary analyses to investigate if the effects of the psychiatric disorders on dementia risk might be explained by exposure to several dementia risk factors that are more common in people with psychiatric disorders, the measures of those exposures may not contain adequate precision or granularity. This means we cannot confidently exclude a role for the exposures tested nor a role for exposures that we did not test.

Fifth, due to the limited data on medication history in the UK Biobank, we have not considered the role of pharmacological treatments of psychiatric disorders, for example, long-term antipsychotic or antidepressant, which may impact on long-term cognitive health.^29^

Sixth, we examined only a limited set of potential disease risk modifiers selected to broadly represent key modifiable dementia risk factors. This list is not exhaustive and does not include environmental factors that may be linked to both psychiatric disorder and dementia, for example, social isolation and sleep disturbance.^30^ Incorporating more detailed variables from clinical records and/or self-report would introduce other sources of bias, including non-random missingness and under-reporting, which tends to be greater among individuals with more severe mental conditions.

Finally, our model and tests of liability are based on a liability threshold model which is an additive model.

In conclusion, our findings provide insights into the complex relationships between dementia, non-affective psychosis and depression. The association cannot be fully explained by psychiatric disorders manifesting as prodromal symptoms of dementia, nor does it appear that it is explained by shared underlying trait liability. Rather, our findings point to the important conclusion that increased rates of dementia in those with psychotic disorders and depression are a consequence of manifesting those disorders. The most likely explanation is that people with psychiatric disorders are associated with greater exposure to additional unidentified dementia risk factors. The identification of these, particularly those that may be modifiable, including the effects of treatment, should now be an important topic for further investigation.

## Supplementary material

10.1136/bmjment-2025-302424 online supplemental file 1

## Data Availability

Data may be obtained from a third party and are not publicly available.
