# Supplementary material for: Genetic investigation of non-affective psychosis and depression as causal risk factors for dementia
Source: BMJ Ment Health. 2026 Jun 24;29(1):e302424. doi: 10.1136/bmjment-2025-302424 (PMC13295947; doi:10.1136/bmjment-2025-302424)
Supplement: online supplemental file 1 [file bmjment-29-1-s001.docx]

**Supplementary Table 1.** Codes used for deriving diagnoses.

| Diagnostic group | Diagnosis | ICD-10 code(s) | UK Biobank variable ID* |
| --- | --- | --- | --- |
| Dementia | Alzheimer's disease |  | 42020 |
|  | Vascular dementia |  | 42022 |
|  | Unspecified dementia** |  | 42018 |
|  | Parkinson’s Disease and dementia in other diseases |  | 42032 and 42018 |
| Psychosis | Schizophrenia | F20 |  |
|  | Delusional disorder | F22 |  |
|  | Acute and transient psychotic disorders | F23 |  |
|  | Schizoaffective disorder | F25 |  |
|  | Unspecified nonorganic psychosis | F29 |  |
| Depression | Single depressive episode | F32 |  |
|  | Recurrent depressive episode | F33 |  |
|  | Persistent mood affective disorders | F34 |  |
|  | Unspecified mood disorder | F39 |  |
| Mania/Bipolar | Mania | F30 |  |
|  | Bipolar disorder | F31 |  |
| Anxiety | Phobic anxiety disorders | F40 |  |
|  | Generalised anxiety disorder | F41 |  |

* - The dementia diagnoses are part of a ‘dementia outcomes’ set of variables defined by the UK Biobank.

** - Defined by the UK Biobank variable ID “42018” excluding Alzheimer's disease, Vascular dementia, Parkinsons disease, Amyotrophic lateral sclerosis and Frontotemporal dementia

**Supplementary Table 2.** Distribution of confounders.

|  | UKB code | In people with Psychosis | In people with Depression | In people  without Psychiatric Diagnosis | In people with Dementia | In people without Dementia |
| --- | --- | --- | --- | --- | --- | --- |
| Smoking: N (% of smokers ever) | 20116 | 1,043 (56.1%) | 27,050 (51.8%) | 145,610 (44.2%) | 4,229 (53.6%) | 178,083 (45.2%) |
| BMI: Mean [SD] | 21001 | 28.3 [5.5] | 28.3 [5.4] | 27.3 [4.6] | 27.8 [4.8] | 27.4 [4.8] |
| Addiction to alcohol: N (% of addicted) | 20406 | 19 (1.01) | 838 (1.6%) | 1,889 (0.57%) | 24 (0.3%) | 2,903 (0.7%) |
| Addiction to any substance or behaviour: N (% of addicted) | 20401 | 40 (2.14%) | 1,774 (3.4%) | 5,204 (1.6%) | 55 (0.7%) | 7,412 (1.9%) |
| Hypertension: N (%) | 131286* | 929 (49.6%) | 25,152 (47.9%) | 128,346 (38.8%) | 5,230 (65.9%) | 157,872 (40.0%) |
| Educational attainment | 6138** | See supplementary Figure 1: Distribution of Educational attainment. | | | | |

* identified by the date of first reported: essential (primary) hypertension

** Coded for the highest of listed by the individual’s achievements as: 6 - College/University degree, 5 – A/AS levels or equivalent, 4 – O levels/GCSE or equivalent, 3 – CSEs or equivalent, 2 – NVQ/HND/HNC or equivalent, 1 – none of the above, NA - other professional qualifications e.g. nursing or teaching and prefer not to answer.

**Supplementary Table 3.** Association of AD.PRS with non-affective psychosis or depression at different cut-off for the age at first record of psychiatric disorder (logistic regression, adjusted for sex)

**(A)**compared to people with dementia without the diagnosis specified in the first row

|  | Psychosis | | | | Depression | | | |
| --- | --- | --- | --- | --- | --- | --- | --- | --- |
| AAFR* cut-off | N with Psychosis | N without Psychosis | B [95%CI] | p | N with Depression | N without Depression | B [95%CI] | p |
| ≤50 | 29 | 7731 | -0.21  [-0.54,0.12] | 0.215 | 431 | 5794 | -0.16  [-0.25,-0.07] | 0.00035 |
| ≤55 | 33 | 7731 | -0.23  [-0.54,0.08] | 0.150 | 552 | 5794 | -0.16  [-0.23,-0.08] | 0.00012 |
| ≤60 | 46 | 7731 | -0.27  [-0.54,-0.004] | 0.046 | 730 | 5794 | -0.15  [-0.22,-0.08] | 3.5x10^-5^ |
| ≤65 | 56 | 7731 | -0.29  [-0.54,-0.05] | 0.0178 | 937 | 5794 | -0.12  [-0.18,-0.05] | 0.00024 |
| ≤70 | 70 | 7731 | -0.21  [-0.42,0.008] | 0.0596 | 1154 | 5794 | -0.12  [-0.17,-0.06] | 6.2x10^-5^ |

**(B)**compared to people with dementia without any psychiatric diagnoses

|  | Psychosis | | | | Depression | | | |
| --- | --- | --- | --- | --- | --- | --- | --- | --- |
| AAFR* cut-off | N with Psychosis | N without any psychiatric diagnoses | B [95%CI] | p | N with Depression | N without any psychiatric diagnoses | B [95%CI] | p |
| ≤50 | 29 | 5057 | -0.23  [-0.56,0.10] | 0.1687 | 431 | 5057 | -0.16  [-0.25,-0.08] | 0.00028 |
| ≤55 | 33 | 5057 | -0.25  [-0.56,0.06] | 0.1129 | 552 | 5057 | -0.16  [-0.24,-0.08] | 9.3x10^-5^ |
| ≤60 | 46 | 5057 | -0.29  [-0.56,-0.03] | 0.0307 | 730 | 5057 | -0.15  [-0.22,-0.08] | 2.6x10^-5^ |
| ≤65 | 56 | 5057 | -0.31  [-0.55,-0.07] | 0.0108 | 937 | 5057 | -0.12  [-0.18,-0.06] | 0.00017 |
| ≤70 | 70 | 5057 | -0.23  [-0.44,-0.01] | 0.0364 | 1154 | 5057 | -0.12  [-0.18,-0.06] | 4.4x10^-5^ |

* AAFR - Age at first report.

**Supplementary Table 4.** Distribution of types of dementia in people with a diagnosis of psychosis or depression (age at first record below 65 years) and in those without such diagnoses.

|  | N (%) | | AD.PRS | | N (%) | | AD.PRS | |
| --- | --- | --- | --- | --- | --- | --- | --- | --- |
|  | With Psychosis | Without Psychosis | B [95%CI] | p | With Depression | Without Depression | B [95%CI] | p |
| Alzheimer’s disease | 15 (1.1) | 2971 (0.74) | -0.28 [-0.9,0.3] | 0.346 | 305 (0.70) | 2320 (0.66) | -0.07 [-0.2,0.0] | 0.226 |
| Parkinson’s Disease with dementia | 7 (0.53) | 704 (0.18) | NA* | NA | 70 (0.16) | 522 (0.15) | -0.09 [-0.3,0.2] | 0.510 |
| Vascular Dementia | 9 (0.68) | 1204 (0.30) | NA* | NA | 163 (0.38) | 867 (0.25) | -0.12 [-0.3,0.0] | 0.174 |
| Unspecified Dementia | 21 (1.6) | 2125 (0.53) | -0.09 [-0.6,0.4] | 0.713 | 298 (0.69) | 1576 (0.45) | 0.01 [-0.1,0.1] | 0.855 |
| No Dementia | 1,275 (96.1) | 393504 (98.3) | -0.00 [-0.1,0.1] | 0.993 | 42526 (98.1) | 344855 (98.5) | 0.01 [-0.0,0.0] | 0.074 |

* The estimates are not provided due to the sample size with Psychosis is ≤ 10.

**Supplementary Figure 1.** Distribution of Educational attainment.

**
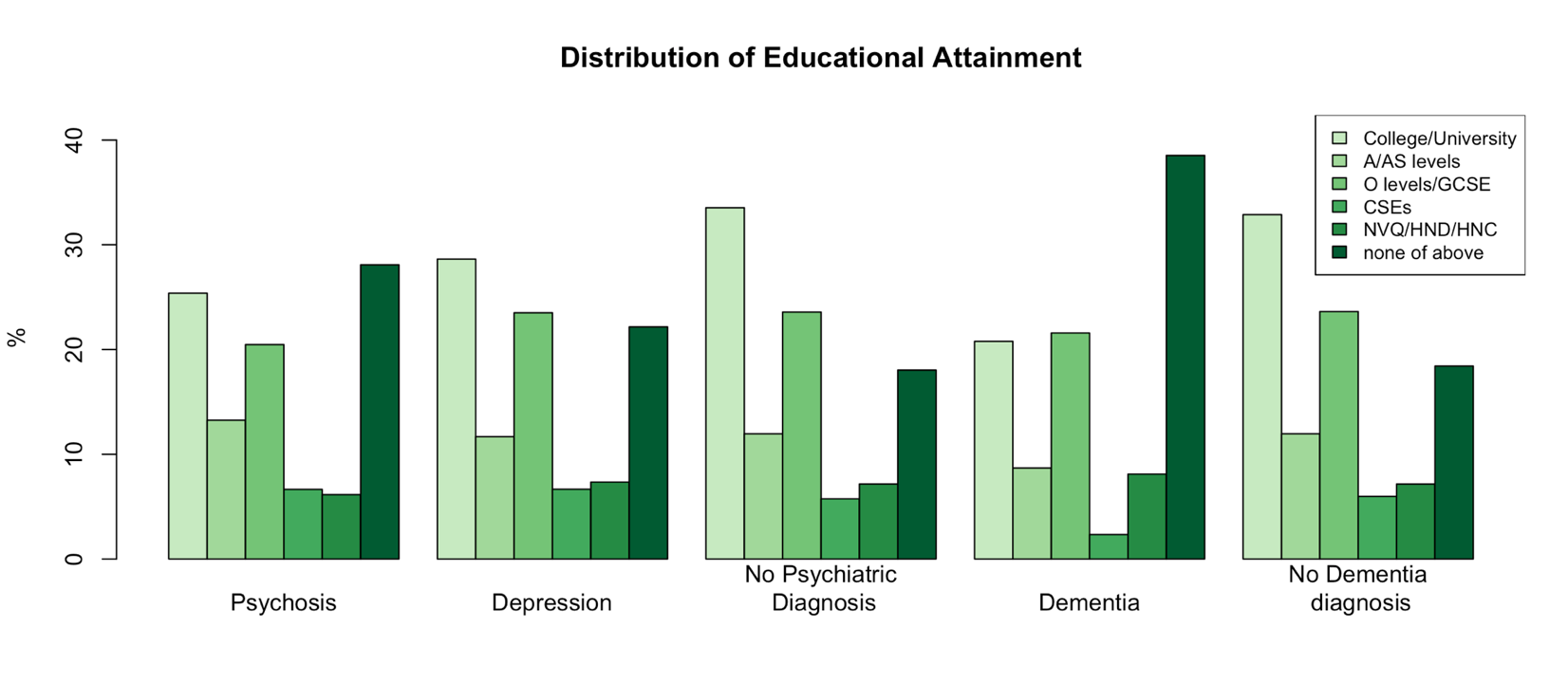
**
